# Persistent inaccuracies in completion of medical certificates of stillbirth: A cross‐sectional study

**DOI:** 10.1111/ppe.12501

**Published:** 2018-10-09

**Authors:** Lucy E. Higgins, Alexander E. P. Heazell, Melissa K. Whitworth

**Affiliations:** ^1^ Faculty of Biology Medicine and Health, Maternal and Fetal Health Research Centre University of Manchester Manchester UK; ^2^ Manchester Academic Health Science Centre St. Mary's Hospital Manchester University NHS Foundation Trust Manchester UK

**Keywords:** accuracy, causal factors, classification, fetal growth restriction, Medical certificate of stillbirth, validity

## Abstract

**Background:**

The UK Medical Certificate of Stillbirth (MCS) records information relevant to the cause of stillbirth of infants ≥24 weeks’ gestation. A cross‐sectional audit demonstrated widespread inaccuracies in MCS completion in 2009 in North West England. A repeat study was conducted to assess whether practice had improved following introduction of a regional care pathway.

**Methods:**

266 MCS issued in 14 North West England obstetric units during 2015 were studied retrospectively. Cause of death was assigned following review of information available at the time of MCS completion. This was compared to that documented on the MCS, and to data from 2009.

**Results:**

Twenty‐three certificates were excluded (20 inadequate data, 3 late miscarriages). 118/243 (49%) MCS contained major errors. Agreement between the MCS and adjudicated cause of stillbirth was fair (Kappa 0.31; 95% CI 0.24, 0.38) and unchanged from 2009 (0.29). In 2015, excluding 34 terminations of pregnancy, the proportion of MCSs documenting “unexplained” stillbirths (113/211; 54%) was reduced compared to 2009 (158/213; 74%); causality could be assigned after case note review in 78% cases. Recognition of fetal growth restriction (FGR) as a cause of stillbirth improved (2015: 30/211; 14% vs 2009: 1/213; 0.5%), although 71% cases were missed. 47% MCSs following termination of pregnancy documented an iatrogenic primary cause of death.

**Conclusions:**

Completion of MCSs remains inaccurate, particularly in recognition of FGR as a cause of stillbirth. Detailed case note review before issuing the MCS could dramatically improve the usefulness of included information; evaluation of practitioner education programmes/internal feedback systems are recommended.

## 1 INTRODUCTION

In the United Kingdom (UK), the Medical Certificate of Stillbirth is issued to the parent(s) of a baby born without signs of life after 24 completed weeks’ gestation (see Figure S1 for an example).^1^ Similar certification systems are used in high‐income countries including the United States of America (USA), Australia, New Zealand, and Ireland. The document is issued within days of birth, recording information relevant to the baby's demise. Hence, information from investigations such as autopsy or placental histopathology is not available at the time of issue. Unlike neonatal, child and adult deaths, in which a Coroner can instigate measures to determine the cause of death where unclear, stillbirths are not currently governed by the UK Coronial law, although this is being reviewed.^2^

Autopsy and placental histopathology can reveal the cause of stillbirth, or provide additional information that alters the management of the mother's future pregnancies/health in up to 50% of cases.^3^ Due to incomplete uptake of these investigations (autopsy: 48.1%, placental histology: 88.8%),^4^ for many parents the Medical Certificate of Stillbirth is the principal source of information regarding the cause of their baby's death. From a public health perspective, the UK Office of National Statistics uses data from Medical Certificates of Stillbirth when describing key characteristics of stillbirths nationally. These are used to assist Governmental evidence‐based policy decisions.^5^ In 2016, the UK Government committed to halving stillbirth rates by 2025.^6^ Therefore, the accuracy of data provided by Medical Certificates of Stillbirth is increasingly important to identify appropriate interventions to prevent avoidable stillbirths.

We conducted a regional cross‐sectional audit of the accuracy of Medical Certificate of Stillbirth completion in the North West England in 2009.^7^ This demonstrated widespread inaccuracies in Medical Certificate of Stillbirth completion (78%), particularly a failure to document fetal growth restriction (FGR) as a cause of death (accounting for ~50% of all stillbirths classified as “unexplained” on the Medical Certificate of Stillbirth). After dissemination of the previous audit findings, and initiatives targeted at improved detection of FGR,^8^, ^9^, ^10^ the accuracy of Medical Certificates of Stillbirth was reassessed. We hypothesised that accuracy would have improved, particularly with respect to recognition and reporting of FGR as a cause of stillbirth.

## 2 METHODS

This study was a cross‐sectional retrospective audit of all Medical Certificates of Stillbirth issued from consultant‐led obstetric units in the North West of England during 2015, using the same methodology as the previous study.^7^ Infants born without signs of life after at least 24 completed weeks’ gestation were identified from birth registers and Medical Certificate of Stillbirth stubs (a brief summary of Medical Certificate of Stillbirth data retained by the hospital after issuing the Medical Certificate of Stillbirth itself). Under UK law, stillbirths resulting from termination of pregnancy at more than 24 weeks’ gestation are also registered as stillbirths.

A standardised electronic data collection pro forma, based on the regional tertiary obstetric unit perinatal case summary data collection tool,^7^ was devised (collecting more maternal and fetal data compared with that used by Cockerill et al) and piloted by one individual (LH) at a single unit. After refinement to the pro forma, 15 trainees in Obstetrics and Gynaecology were trained to extract anonymised data from the medical records including copies of the Medical Certificate of Stillbirth (if available) or data from Medical Certificate of Stillbirth stubs (where unavailable). All units that participated in the previous audit, as well as two additional units, agreed to participate (N = 14 after local reorganisation of maternity services).

Detailed case note review was performed, including assessment of maternal characteristics (ethnicity, height, weight, age, parity, use of cigarettes, alcohol or recreational drugs), fetal gender, and gestation at diagnosis of fetal death in utero, fetal growth trajectory (from ultrasound scan or symphysiofundal height) as customised to maternal characteristics,^11^ details of the presenting complaint, date of fetal death in utero diagnosis, details of the fetus/placenta at delivery including birth and placental weights and the presence of visible congenital abnormalities. Maternal haematological, biochemical, microbiological, virology, and immunology results from the pregnancy and immediate postpartum period (reported prior to the completion of the Medical Certificate of Stillbirth) were also collected. Any results available after this time (such as fetal autopsy or placental histology) were not included.

To minimise inflation of gestational age contributing to over‐diagnosis of FGR, the birthweight centile, adjusted for maternal characteristics was calculated based on the date fetal demise was confirmed. In accordance with a recent international consensus statement, FGR was considered to be present if the baby's birthweight was <3^rd^ customised centile, or two or more of the following were present: (a) AC/EFW <10^th^ centile; (b) AC/EFW crossing >2 quartiles on non‐customized growth centiles; (c) Cerebral perfusion ratio <5^th^ centile or umbilical artery pulsatility index >95^th^ centile.^13^ In addition, the diagnosis of FGR at borderline centiles (3‐10) was strengthened by evidence of placental insufficiency (abnormal fetoplacental Doppler results, ultrasound confirmed oligohydramnios or a markedly small^13^ or visibly grossly infarcted placenta).^14^ Where birthweight was >3^rd^ and <10^th^ centile but did not fulfil the criteria above an alternative diagnosis was assigned where judged to be clinical significant eg placental abruption, chorioamnionitis, and intrapartum hypoxia.

Data extractors were provided with a copy of the ReCoDe classification of stillbirth.^15^ This classification was chosen for i) consistency with the previous study, ii) low rate of “unexplained” stillbirths, and iii) recognition of FGR as a primary cause of death. The cause of stillbirth was then assigned by reviewers according to ReCoDe categories, based only on information that was available at the time of Medical Certificate of Stillbirth completion. For example, autopsy or placental histopathology results (usually unavailable for up to 6 weeks after birth) were not taken into account. As ReCoDe is a hierarchical classification system this promotes the importance of some abnormalities (eg FGR) over other potentially causal events eg intrapartum hypoxia. However, data extractors were asked to consider the relevant contributions of competing relevant conditions at death, rather than obeying the strict hierarchy of described conditions. For example, where FGR was noted in the presence of an alternative potential cause of death (for example significant placental abruption), data extractors were asked to consider which was likely to have been the main condition leading to fetal demise. Furthermore, secondary analysis using the ReCoDe‐R system was also performed.^16^ This demotes FGR in the hierarchy of relevant conditions at death to prioritise other identifiable causes and has been shown to more than halve the proportion of stillbirths attributed to FGR.

Where termination of pregnancy had occurred, data extractors were instructed to assign an iatrogenic primary cause of death (as medical intervention directly led to the fetus’ demise at that specific point in time), with recognition of the condition leading to the decision for termination to be acknowledged as a secondary cause, under either “other diseases or conditions in fetus” or “other maternal diseases or conditions affecting fetus” (Figure S1).

### 2.1 Statistical analysis

Data for gestation at stillbirth and primary cause of stillbirth were compared between that documented on the Medical Certificate of Stillbirth and that expected from case note review. Accuracy was quantified using the Kappa statistic; agreement was categorised as nil (0), slight (0.1‐0.20), fair (0.21‐0.40), moderate (0.41‐0.60), substantial (0.61‐0.80), or almost complete (0.81‐1.0).^17^ Errors in Medical Certificate of Stillbirth completion were categorised as previously,^7^ adapting the Pritt et al^18^ template for medical certificates of adult death. I and II indicated minor errors (inaccurate gestational age, autopsy consent status, and minor missed maternal co‐morbidities not contributing to the cause of death eg mild asthma). III and IV indicated major inaccuracies/omissions that would alter how the Medical Certificate of Stillbirth was interpreted by “the family, other physicians, or personnel gathering health statistics.” Examples of this included issuing a Medical Certificate of Stillbirth for an infant miscarried before 24 weeks’ gestation, failure to reflect iatrogenic death in terminations of pregnancy, failure to recognise FGR, significant contributory maternal health conditions such as pre‐eclampsia, or stating “unexplained” when an obvious cause could be identified.

Changes in the prevalence and documentation of different primary causes of stillbirth between 2009 and 2015, and between 2015 Medical Certificate of Stillbirth‐documented and adjudicated cause of stillbirth were assessed.

## 3 RESULTS

Data were received from all obstetric units approached. 266 Medical Certificates of Stillbirth were issued in the 12‐month audit period. Three excluded certificates were issued for fetuses known to have died prior to 24 completed weeks’ gestation. In five cases, insufficient data was accessible to ascertain correct gestation at stillbirth, in 14 cases the Medical Certificate of Stillbirth documented cause of stillbirth could not be confirmed and in one final case the maternal medical records could not be accessed. This left 243 cases for analysis (34 terminations of pregnancy).

Table 1 presents the characteristics of the 243 stillborn infants from 2015. Where directly comparable data are available, there was no difference between included pregnancies in 2009 and 2015 in terms of median BMI (25.4, interquartile range [IQR] 22.6‐29.6) vs 25.3 (IQR 21.6‐30.4), ETHNICITY (Caucasian 151/213 [71%] vs 160/241 [66%]), median parity (1, IQR 0‐2) vs (1, IQR 0‐2), smoker status 56/213 (26%) vs 51/241 (21%), median gestation at diagnosis (34^+2^ weeks [IQR 28^+3^‐38^+2^] vs 32^+3^ weeks [IQR 26^+5^‐37^+6^]), or median birthweight (1815 g [IQR 886‐2750] vs 1480 g [IQR 708‐2753]).

**Table 1:** Characteristics of the cohort

Maternal characteristics	
Age (years)	30 (26‐35)
BMI (kg/m^2^)	25.4 (21.6‐30.3)
Ethnicity	
Caucasian	160/241 (66.4%)
Black	22/241 (9.1%)
Asian	46/241 (19.1%)
Other	11/241 (4.6%)
Parity	1 (0‐2)
Smoker	51/241 (21.2%)
Continued alcohol intake	6/240 (2.5%)
Recreational drug misuse	5/227 (2.2%)

Birth outcome	
Termination of pregnancy	34/243 (14.0%)
Timing in relation to labour	
Antepartum	211/243 (86.8%)
Intrapartum	27/243 (11.1%)
Unknown	5/243 (2.1%)
Gestation at diagnosis (weeks^+days^)	32^+3^ (26^+4^‐37^+6^)
Diagnosis to delivery interval (days)	1 (0‐2)
Male	136/242 (56.2%)
Birthweight	1480 (708‐2753)
Birthweight centile	9.4 (0.3‐42.3)

Compared with regional demographics, mothers of stillborn infants were more likely to be of Asian (Indian, Pakistani, or Bangladeshi) heritage and to smoke. The distribution of birth weights was skewed with a high preponderance of small for gestational age babies. Key: BMI = body mass index. Data are shown as median (interquartile range) or number (percentage).

A highly skewed birthweight distribution is seen in both study populations; although median birthweight centile was higher in 2015 (9.4, IQR 0.3‐42.3) compared to 2009 (4, IQR 0.0‐37.5). In 2015, 51% (123/243), stillborn fetuses had a birthweight <10^th^ centile, with 38% (n = 91) at <3^rd^ centile. Only 6% had a birthweight >90^th^ centile. 6% (11/196) completed growth charts showed suspected small for gestational age (SGA) or static symphysio‐fundal height. Of 113 infants who had at least one growth scan, 23% (n = 26) were SGA with a further 8% (n = 9) demonstrating poor growth trajectory. Where documented, umbilical artery Doppler abnormalities and oligohydramnios were present in 11% (21/190) and 11% (22/208) cases, respectively.

The majority of stillbirths in 2015 were diagnosed antepartum (Table 1) with an interval from diagnosis of stillbirth to delivery ranging from 0 to 64 days. Autopsy consent status at the time of Medical Certificate of Stillbirth completion was ascertained in 237 cases in 2015; 35% (n = 83) had consented to autopsy.

### 3.1 Trends in causes of stillbirth as documented and adjudicated

Between 2009 and 2015, there was an increase in reporting of fetal causes (predominantly FGR) and decrease in reporting of “unexplained” causes on the Medical Certificate of Stillbirth (Table 2). However, these causes remained under‐ and over‐reported, respectively. Other broad categories of cause of stillbirth remained stable except for an increase in reporting of intrapartum asphyxia (55%, 11/20 of stillbirths in one hospital, of which 64%, [7/11] cases were adjudicated to be due to FGR).

**Table 2:** : Comparison of primary cause of stillbirth (excluding stillbirths resulting from termination of pregnancy) as recorded on the Medical Certificate of Stillbirth compared with adjudicated cause of death for 2009 and 2015

Classification		2009				2015			
		MCS		Adjudicated		MCS		Adjudicated	
		N	% (95% CI)	N	% (95% CI)	N	% (95% CI)	N	% (95% CI)
A1	Lethal fetal abnormality	20	9.4 (6.2, 14.1)	20	9.4 (6.2, 14.1)	11	5.2 (2.8, 9.1)	17	8.1 (5.1, 12.5)
A2	Infection	0	0 (0.0, 1.8)	0	0 (0.0, 1.8)	0	0 (0.0, 1.8)	2	1.0 (0.3, 3.4)
A3	Non‐immune hydrops	1	0.5 (0.1, 2.6)	1	0.5 (0.1, 2.6)	3	1.4 (0.5, 4.1)	2	1.0 (0.3, 3.4)
A5	Fetomaternal hydrops	1	0.5 (0.1, 2.6)	2	0.9 (0.3, 3.4)	0	0 (0.0, 1.8)	2	1.0 (0.3, 3.4)
A6	Twin‐twin transfusion	0	0 (0.0, 1.8)	7	3.3 (1.6, 6.6)	4	1.9 (0.7, 4.8)	5	2.4 (1.0, 5.4)
A7	Fetal growth restriction	1	0.5 (0.1, 2.6)	94	44.1 (37.6, 50.8)	30	14.2 (10.2, 19.6)	97	46.0 (39.4, 52.7)
A8	Other (fetal)	2	0.9 (0.3, 3.4)	9	4.2 (2.2, 7.8)	1	0.5 (0.1, 2.6)	1	0.5 (0.1, 2.6)
**A**	**Total Fetal**	**25**	**11.7 (8.1, 16.8)**	**133**	**62.4 (55.8, 68.7)**	**49**	**23.2 (18.0, 29.4)**	**129**	**61.1 (54.4, 67.5)**
B1	Cord prolapse	0	0 (0.0, 1.8)	2	0.9 (0.3, 3.4)	2	1.0 (0.3, 3.4)	2	1.0 (0.3, 3.4)
B2	Constricting loop/knot	4	1.9 (0.7, 4.7)	0	0 (0.0, 1.8)	5	2.4 (1.0, 5.4)	7	3.3 (1.6, 6.7)
**B**	**Total Umbilical Cord**	**4**	**1.9 (0.7, 4.7)**	**2**	**0.9 (0.3, 3.4)**	**7**	**3.3 (1.6, 6.7)**	**9**	**4.3 (2.3, 7.9)**
C1	Placental abruption	17	8.0 (5.0, 12.4)	18	8.5 (5.4, 13.0)	13	6.2 (3.6, 10.3)	16	7.6 (4.7, 12.0)
C2	Placenta praevia	0	0 (0.0, 1.8)	0	0.0 (0.0, 1.8)	0	0 (0.0, 1.8)	1	0.5 (0.1, 2.6)
C3	Vasa praevia	1	0.5 (0.1, 2.6)	0	0.0 (0.1, 1.8)	0	0 (0.0, 1.8)	1	0.5 (0.1, 2.6)
C4	Placental insufficiency	0	0 (0.0, 1.8)	4	1.9 (0.7, 4.7)	0	0 (0.0, 1.8)	2	1.0 (0.3, 3.4)
C5	Other (placenta)	1	0.5 (0.1, 2.6)	0	0 (0.0, 1.8)	2	1.0 (0.3, 3.4)	0	0 (0.0, 1.8)
**C**	**Total Placental**	**19**	**8.9 (5.8, 13.5)**	**22**	**10.3 6.9, 15.1)**	**15**	**7.1 (4.4, 11.4)**	**20**	**9.5 (6.2, 14.2)**
D1	Chorioamnionitis	2	0.9 (0.3, 3.4)	3	1.4 (0.5, 4.1)	3	1.4 (0.5, 4.1)	6	2.8 (1.3, 6.1)
D2	Oligohydramnios	0	0 (0.0, 1.8)	0	0 (0.0, 1.8)	0	0 (0.0, 1.8)	0	0 (0.0, 1.8)
D3	Polyhydramnios	0	0 (0.0, 1.8)	0	0 (0.0, 1.8)	0	0 (0.0, 1.8)	1	0.5 (0.1, 2.6)
D4	Other (amniotic fluid)	0	0 (0.0, 1.8)	0	0 (0.0, 1.8)	1	0.5 (0.1, 2.6)	0	0 (0.0, 1.8)
**D**	**Total Amniotic Fluid**	**2**	**0.9 (0.3, 3.4)**	**3**	**1.4 (0.5, 4.1)**	**4**	**1.9 (0.7, 4.8)**	**7**	**3.3 (1.6, 6.7)**
E1	Uterine rupture	1	0.5 (0.1, 2.6)	1	0.5 (0.1, 2.6)	0	0 (0.0, 1.8)	1	0.5 (0.1, 2.6)
E2	Uterine abnormalities	0	0 (0.0, 1.8)	0	0 (0.0, 1.8)	1	0.5 (0.1, 2.6)	1	0.5 (0.1, 2.6)
**E**	**Total Uterine**	**1**	**0.5 (0.1, 2.6)**	**1**	**0.5 (0.1, 2.6)**	**1**	**0.5 (0.1, 2.6)**	**2**	**1.0 (0.3, 3.4)**
F1	Diabetes	1	0.5 (0.1, 2.6)	8	3.8 (1.9, 7.2)	1	0.5 (0.1, 2.6)	6	2.8 (1.3, 6.1)
F4	Hypertensive diseases in pregnancy	0	0 (0.0, 1.8)	1	0.5 (0.1, 2.6)	0	0 (0.0, 1.8)	2	1.0 (0.3, 3.4)
F5	Antiphospholipid syndrome	0	0 (0.0, 1.8)	0	0 (0.0, 1.8)	0	0 (0.0, 1.8)	1	0.5 (0.1, 2.6)
F6	Cholestasis	2	0.9 (0.3, 3.4)	1	0.5 (0.1, 2.6)	0	0 (0.0, 1.8)	0	0 (0.0, 1.8)
F8	Other (maternal)	0	0 (0.0, 1.8)	0	0 (0.0, 1.8)	2	1.0 (0.3, 3.4)	4	1.9 (0.7, 4.8)
**F**	**Total Maternal**	**3**	**1.4 (0.5, 4.1)**	**10**	**4.7 (2.6, 8.4)**	**3**	**1.4 (0.5, 4.1)**	**13**	**6.2 (3.6, 10.3)**
G1	Asphyxia	1	0.5 (0.1, 2.6)	2	0.9 (0.3, 3.4)	12	5.7 (3.3, 9.7)	4	1.9 (0.7, 4.8)
G2	Birth trauma	0	0 (0, 1.8)	1	0.5 (0.1, 2.6)	0	0 (0.0, 1.8)	0	0 (0.0, 1.8)
**G**	**Total Intrapartum**	**1**	**0.5 (0.1, 2.6)**	**3**	**1.4 (0.5, 4.1)**	**12**	**5.7 (3.3, 9.7)**	**4**	**1.9 (0.7, 4.8)**
H2	Iatrogenic trauma	0	0 (0.0, 1.8)	0	0 (0.0, 1.8)	2	1.0 (0.3, 3.4)	34	16.1 (11.8, 21.7)
**H**	**Total Traumatic**	**0**	**0.0 (0, 1.8)**	**0**	**0 (0.0, 1.8)**	**2**	**1.0 (0.3, 3.4)**	**0**	**0 (0.0, 1.8)**
I1	No relevant condition identified	125	58.7 (52.0, 65.1)	38	17.8 (13.3, 23.5)	112	53.1 (46.4, 59.7)	21	10.0 (6.6, 14.7)
I2	No information available	33	15.5 (11.3, 21.0)	1	0.5 (0.1, 2.6)	1	0.5 (0.1, 2.6)	8	3.8 (1.9, 7.3)
**I**	**Total Unexplained**	**158**	**74.2 (67.9, 79.6)**	**39**	**18.3 (13.7, 24.1)**	**113**	**53.6 (46.8, 60.2)**	**29**	**13.7 (9.7, 19.0)**

Causes are categorised according to the Relevant Condition at Death (ReCoDe) classification system.^14^ Although fetal growth restriction (FGR) is being acknowledged as a cause of stillbirth more frequently than in 2009, there is a persistent failure to identify all cases of stillbirth primarily due to FGR. Furthermore, inappropriate classification of stillbirths as “unexplained” (equivalent to classifications I1 and I2) persists, although at a lower rate than in 2009. There was an increase in the reported rate of asphyxia as primary cause of stillbirth between 2009 and 2015, resulting in an increase in the proportion of stillbirths being classified as “intrapartum.” On review, the majority of these stillbirths occurred in compromised, FGR babies. Key: MCS = Medical Certificate of Stillbirth, Adjudicated = adjudicated cause of stillbirth after review of medical records.

### 3.2 Accuracy of Medical Certificate of Stillbirth completion

Major errors in Medical Certificate of Stillbirth completion were present in 49% (121/246) cases. These almost universally comprised inappropriate classification as “unexplained” (98%, 116/121), within which the single most frequent missed cause was FGR (47%, 55/116). Three major errors resulted from inappropriately issuing Medical Certificates of Stillbirth to parents of miscarried (<24 weeks gestation) infants. Minor errors were found in a further 25% (61/243) cases. It was not possible to assess differences in accuracy of Medical Certificates of Stillbirth completion in relation to profession of the issuer (midwife or doctor); where identifiable the vast majority (88%, 175/199) were issued by midwives. There was no difference for either major or minor errors according to ethnic category (data not shown).

The Kappa statistic for gestation was 0.67 (95% confidence interval [CI] 0.59, 0.73), indicating substantial agreement. This was substantially lower than in the previous audit (0.88). The Kappa statistic for cause of death in non‐termination of pregnancy stillbirths was 0.31 (95% CI 0.24, 0.38) indicating fair agreement. This was comparable to the findings of the previous audit (0.29; Table 2). However, some improvement was seen. The most commonly documented classification of the cause of death on Medical Certificate of Stillbirth in both audits was “unexplained” (equivalent to ReCoDe classification I1 and I2), but this decreased in 2015. Yet, in 74% (84/113) of 2015 cases initially classified as “unexplained,” a cause was found on case review (no significant difference compared to 2009).

In 2015, 30/211 (14%; 95% CI 10.2, 19.6) stillbirths were originally classified on the Medical Certificate of Stillbirth as due to FGR; two were adjudicated to be primarily due to other causes (congenital abnormality and placental abruption; although FGR/SGA was also present). This contrasts with just 0.5% (95% CI 0.1, 2.6; 1/213) in 2009. After adjudication, FGR was determined the primary cause of death in 46% (95% CI 39.4, 52.7; 97/211) infants in 2015 vs 44% (95% CI 37.6, 50.8; 94/213) in 2009. Using the ReCoDe‐R classification this was reduced to 23% (95% CI 17.6, 28.9; 48/211) in 2015 although it remained the predominant cause of stillbirth within the study population, with 37% (95% CI 24.7, 50.7; 18/49) reclassified stillbirths being attributed to placental insufficiency (Table S1).

In the 34 terminations of pregnancy in 2015, 47% (n = 16) Medical Certificates of Stillbirth reflected the iatrogenic nature of the infant's death; 44% (n = 15) were erroneously classified as being primarily caused by congenital abnormality that instigated the decision for termination.

## 4 COMMENT

### 4.1 Principal findings

In this follow‐up study, we have shown that although there has been modest improvement, the previously reported inaccuracies in Medical Certificates of Stillbirth have persisted.^7^ This reinforces the need for an ongoing perinatal surveillance process, as conclusions drawn from Medical Certificate of Stillbirth data alone inadequately report the true incidence of potentially avoidable causes of stillbirth, such as FGR. In this, and the preceding study, FGR has been determined to be the most common adjudicated cause of stillbirth.

### 4.2 Strengths of the study

The major strength of this study is the systematic examination of all stillbirths from a variety of hospital settings. The total identified cases in the audit period matches that reported to the MBRRACE‐UK perinatal surveillance programme from constituent hospitals in the same time period;^4^ 92.0% of these cases were reviewed in detail. Secondly, we demonstrate that even with minimal specific training clinicians can extract appropriate data to reach an informed conclusion regarding cause of stillbirth. Finally, by applying the same methodology in two consecutive studies, we are able to assess changes over time in the reporting, and prevalence, of various causes of stillbirth.

### 4.3 Limitations of the data

The major weakness is that we were unable to examine the accuracy of secondary information included on the Medical Certificate of Stillbirth. This could be improved by routine inclusion of a photocopy of the Medical Certificate of Stillbirth in the maternal notes. Secondly, it was necessary to exclude 23 cases from the study population. This may have minimised the reported error rate.

Another source of bias is likely to arise from use of date of confirmation of fetal death as a proxy for the date of actual fetal death within the study. If this could be determined, it is likely that the accuracy of reported gestational age would be worse, and that the proportion of FGR‐attributed deaths in the “borderline” cases (centiles 5‐10) minimised, although steps were taken to reinforce the diagnosis in these cases. No gold‐standard method for ascertaining actual date of fetal death is described; indeed one proposed algorithm could only be implemented in 47% of cases in the author's own study.^19^ Thus date of confirmation of fetal death is the only fixed time point that could be used.

Finally, use of the ReCoDe classification system,^15^ with its hierarchical design, to assign cause of fetal death may have influenced the proportion of stillbirths attributed to FGR.^16^ Over 31 classification systems for cause of stillbirth exist, and cause of death for an individual infant may differ by classification system.^20^ Use of the Cause Of Death and Associated Conditions (CODAC) system^21^ has been promoted following comparison of six major classification systems.^22^ However, both ReCoDe and CODAC have a similarly low rate of “unexplained” stillbirths (14% and 10%, respectively) and both consider FGR an independent cause of stillbirth. ReCoDe is the only system that has been developed specifically for classification of cause of stillbirth and enables better comparison of the trends in causes of stillbirth between the two studies.

### 4.4 Interpretation

Although some progress has been made (reduction in the proportion of Medical Certificates of Stillbirth with “unexplained” causes and improvement in baseline recognition of FGR) it is concerning that in the last 6 years there has been no significant improvement in agreement between initial and reviewed causes of stillbirth, with a similar proportion of major errors, and a significant burden of undocumented/unrecognised FGR. This is despite regional,^8^ national,^9^ and international^10^ initiatives focussed on the antenatal detection and management of FGR. In one participating unit, the mechanism of death (perinatal asphyxia) was substituted for the cause (FGR); while both may reflect underlying placental insufficiency, overlooking FGR in this context may adversely affect care in a subsequent pregnancy.

The inaccuracy of documented gestational age was affected by use of delivery date (rather than the date on which fetal death was confirmed) to calculate gestational age at stillbirth, an error also noted in the USA.^23^ This was particularly influential where co‐twin survival resulted in up to 64 days between confirmation of death and birth. Although not part of the primary analysis, it was concerning that the iatrogenic nature of death is not accurately recorded in almost 50% of termination of pregnancy cases; substitution of the congenital abnormality as the cause of death may lead to an overestimate of this disorder as a cause for stillbirth.

We propose that to improve the accuracy of data reported on Medical Certificates of Stillbirth, several steps are taken. Fundamentally, the Medical Certificate of Stillbirth should not be completed without (ideally multidisciplinary) review of the predisposing factors, pregnancy chronology including growth charts and scan reports, presentations, and postnatal events. This could be combined with rapid case review (now standard practice in many UK hospitals), and with reference to the ReCoDe classification categories (Table 3).

**Table 3:** Suggested use of ReCoDe classification system to aid full completion of Medical Certificates of Stillbirth

A. Fetus	Lethal congenital anomaly Infection: 2.1 Chronic, 2.2 Acute Non‐immune hydrops Iso immunisation Fetomaternal haemorrhage Twin‐twin transfusion Fetal growth restriction	Usually fetal direct (a) Consider fetal indirect (b) & other contributory (e)
B. Umbilical cord	Prolapse Constricting loop or knot Velamentous insertion	Usually fetal direct (a) Usually fetal indirect (b)
C. Placenta	Abruptio Praevia Praevia Vasa praevia Placental insufficiency/infarction	Usually fetal direct (a) May be fetal direct (a) or indirect (b)
D. Amniotic fluid	Chorioamnionitis Oligohydramnios Polyhydramnios	May be fetal direct (a) or indirect (b)
E. Uterus	Rupture	Often maternal direct (c)
F. Mother	Diabetes Thyroid disease Essential hypertension Hypertensive disease in pregnancy Lupus / antiphospholipid syndrome Cholestasis Drug abuse	May be maternal direct (c) Consider maternal indirect (d) and other contributory (e)
G. Intrapartum	Asphyxia Birth trauma	Usually fetal direct (a)
H. Trauma	External Iatrogenic (eg termination of pregnancy)	Usually fetal direct (a) Consider maternal direct (c) or indirect (d)
I. Unclassified	No relevant condition identified No information available	Usually fetal direct (a)

Secondly, standardised training should be provided to all professionals completing Medical Certificates of Stillbirth, particularly in relation to the significance of FGR. From a similar baseline prevalence of major errors,^24^, ^25^, ^26^, ^27^, ^28^, ^29^ education programmes have improved the accuracy of adult medical certificates of death.^24^, ^29^, ^30^, ^31^, ^32^, ^33^, ^34^ Whether such improvements are sustained is unknown, and there is no corresponding evidence in stillbirths.

The results of our study suggest that Medical Certificates of Stillbirth are routinely issued by midwives rather than doctors. The number of Medical Certificates of Stillbirth issued by doctors in this study period was too small to assess whether a difference in accuracy exists between Medical Certificates of Stillbirth issued by midwives and doctors. This question needs addressing as a matter of urgency. Alternatively, completion of Medical Certificates of Stillbirth could be restricted to specifically trained individuals with a specialist interest in bereavement. Maintenance of standards within a small, motivated group is likely to be more achievable but carries significant organisational implications to ensure that one of these select individuals is always available to provide this service.

Finally, internal feedback to the individual issuing the Medical Certificate of Stillbirth, after departmental review could be implemented.^28^ This could be combined with a reissuing of the Medical Certificate of Stillbirth as has been previously suggested.^7^ However, this would require detailed consultation regarding the view of bereaved parents, which to our knowledge has not yet been conducted, and a change in the UK law.

The contemporaneous Midlands and North of England Stillbirth Study (MiNESS)^35^ describes a strikingly similar distribution of causes of death to that described here. This gives confidence in the validity/generalisability of the results presented here. MiNESS reports a greater proportion of stillbirths attributed to placental insufficiency; this is expected given that they had access to placental histopathology data. Thus, we believe it is clear that substantial improvements in the accuracy of Medical Certificates of Stillbirth can be made with structured case review alone.

## 5 CONCLUSIONS

Small improvements have been made in Medical Certificate of Stillbirth accuracy, but further improvement is needed to eliminate persistent inaccuracies. There is no reason to suspect that this is an anomaly limited to the North West of England: additional data are needed from other areas, nationally, and internationally. Reliance on perinatal surveillance reports, rather than statistics obtained from Medical Certificates of Stillbirth is advocated in formation of policies to reduce national and international stillbirth rates. Simple measures, such as staff training in the importance of FGR as a cause of stillbirth, structured multidisciplinary case review and practitioner education/feedback, are likely to improve Medical Certificate of Stillbirth accuracy but require prospective evaluation.
