# Supplementary figures and images for: Persistent inaccuracies in completion of medical certificates of stillbirth: A cross‐sectional study
Source: Paediatr Perinat Epidemiol. 2018 Oct 9;32(5):474–81. doi: 10.1111/ppe.12501 (PMC6221058; doi:10.1111/ppe.12501)

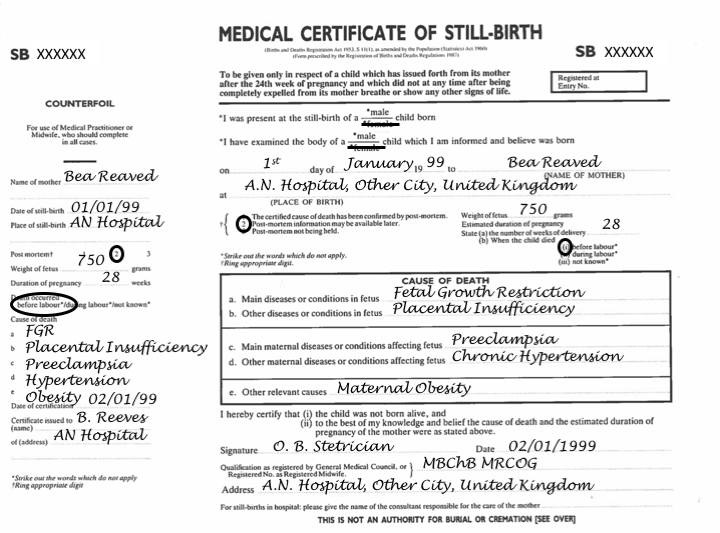

Supplement: Supplementary file 1 [file PPE-32-474-s001.tiff]
