# Supplementary material for: Persistent inaccuracies in completion of medical certificates of stillbirth: A cross‐sectional study
Source: Paediatr Perinat Epidemiol. 2018 Oct 9;32(5):474–81. doi: 10.1111/ppe.12501 (PMC6221058; doi:10.1111/ppe.12501)
Supplement: Supplementary file 2 [file PPE-32-474-s002.docx]

Table S1: Comparison of primary cause of stillbirth (excluding stillbirths resulting from termination of pregnancy) for deaths in 2015 according to ReCoDe and ReCoDe-R classification systems.

| **Classification** | | **ReCoDe** | | | **ReCoDe-R** | | |
| --- | --- | --- | --- | --- | --- | --- | --- |
|  |  | N | % | 95% CI | N | % | 95% CI |
| A1 | Lethal fetal abnormality | 17 | 8.1 | 5.1 – 12.5 | 17 | 8.1 | 5.1 – 12.5 |
| A2 | Infection | 2 | 1.0 | 0.3 – 3.4 | 2 | 1.0 | 0.3 – 3.4 |
| A3 | Non-immune hydrops | 2 | 1.0 | 0.3 – 3.4 | 2 | 1.0 | 0.3 – 3.4 |
| A5 | Fetomaternal hydrops | 2 | 1.0 | 0.3 – 3.4 | 2 | 1.0 | 0.3 – 3.4 |
| A6 | Twin-twin transfusion | 5 | 2.4 | 1.0 – 5.4 | 5 | 2.4 | 1.0 – 5.4 |
| A7 | Fetal growth restriction | 97 | 46.0 | 39.4 – 52.7 | 48 | 22.8 | 17.6 – 28.9 |
| A8 | Other (fetal) | 1 | 0.5 | 0.1 – 2.6 | 1 | 0.5 | 0.1 – 2.6 |
| **A** | **Total Fetal** | **126** | **59.7** | **53.0 – 66.1** | **77** | **36.5** | **30.3 – 43.2** |
| B1 | Cord prolapse | 2 | 1.0 | 0.3 – 3.4 | 3 | 1.4 | 0.5 – 4.1 |
| B2 | Constricting loop/knot | 7 | 3.3 | 1.6 – 6.7 | 12 | 5.7 | 3.3 – 9.7 |
| **B** | **Total Umbilical Cord** | **9** | **4.3** | **2.3 – 7.9** | **15** | **7.1** | **4.4 – 11.4** |
| C1 | Placental abruption | 16 | 7.6 | 4.7 – 12.0 | 25 | 11.9 | 8.2 – 16.9 |
| C2 | Placenta praevia | 1 | 0.5 | 0.1 – 2.6 | 1 | 0.5 | 0.1 – 2.6 |
| C3 | Vasa praevia | 1 | 0.5 | 0.1 – 2.6 | 1 | 0.5 | 0.1 – 2.6 |
| C4 | Placental insufficiency | 2 | 1.0 | 0.3 – 3.4 | 20 | 9.5 | 6.2 – 14.2 |
| C5 | Other (placenta) | 0 | 0.0 | 0.0 – 1.8 | 1 | 0.5 | 0.1 – 2.6 |
| **C** | **Total Placental** | **20** | **9.5** | **6.2 – 14.2** | **48** | **22.8** | **17.6 – 28.9** |
| D1 | Chorioamnionitis | 6 | 2.8 | 1.3 – 6.1 | 10 | 4.7 | 2.6 – 8.5 |
| D2 | Oligohydramnios | 0 | 0.0 | 0.0 – 1.8 | 2 | 1.0 | 0.3 – 3.4 |
| D3 | Polyhydramnios | 1 | 0.5 | 0.1 – 2.6 | 1 | 0.5 | 0.1 – 2.6 |
| D4 | Other (amniotic fluid) | 0 | 0.0 | 0.0 – 1.8 | 0 | 0.0 | 0.0 – 1.8 |
| **D** | **Total Amniotic Fluid** | **7** | **3.3** | **1.6 – 6.7** | **13** | **6.2** | **3.6 – 10.3** |
| E1 | Uterine rupture | 1 | 0.5 | 0.1 – 2.6 | 1 | 0.5 | 0.1 – 2.6 |
| E2 | Uterine abnormalities | 1 | 0.5 | 0.1 – 2.6 | 1 | 0.5 | 0.1 – 2.6 |
| **E** | **Total Uterine** | **2** | **1.0** | **0.3 – 3.4** | **2** | **1.0** | **0.3 – 3.4** |
| F1 | Diabetes | 6 | 2.8 | 1.3 – 6.1 | 8 | 3.8 | 1.9 – 7.3 |
| F3 | Essential hypertension | 0 | 0.0 | 0.0 – 1.8 | 2 | 1.0 | 0.3 – 3.4 |
| F4 | Hypertensive diseases in pregnancy | 2 | 1.0 | 0.3 – 3.4 | 5 | 2.4 | 1.0 – 5.4 |
| F5 | Antiphospholipid syndrome | 1 | 0.5 | 0.1 – 2.6 | 2 | 1.0 | 0.3 – 3.4 |
| F6 | Cholestasis | 0 | 0.0 | 0.0 – 1.8 | 0 | 0.0 | 0.0 – 1.8 |
| F8 | Other (maternal) | 4 | 1.9 | 0.7 – 4.8 | 5 | 2.4 | 1.0 – 5.4 |
| **F** | **Total Maternal** | **13** | **6.2** | **3.6 – 10.3** | **22** | **10.4** | **7.0 – 15.3** |
| G1 | Asphyxia | 4 | 1.9 | 0.7 – 4.8 | 4 | 1.9 | 0.7 – 4.8 |
| G2 | Birth trauma | 0 | 0.0 | 0.0 – 1.8 | 0 | 0.0 | 0.0 – 1.8 |
| **G** | **Total Traumatic** | **4** | **1.9** | **0.7 – 4.8** | **4** | **1.9** | **0.7 – 4.8** |
| I1 | No relevant condition identified | 21 | 10.0 | 6.6 – 14.7 | 21 | 10.0 | 6.6 – 14.7 |
| I2 | No information available | 8 | 3.8 | 1.9 – 7.3 | 8 | 3.8 | 1.9 – 7.3 |
| **I** | **Total Unexplained** | **29** | **13.7** | **9.7 – 19.0** | **29** | **13.7** | **9.7 – 19.0** |

Causes are categorised according to the Relevant Condition at Death (ReCoDe) classification system^14^ and ReCoDe-R system^15^. The ReCoDe-R system demotes fetal growth restriction in the hierarchy of causes of stillbirth to where no other possible cause of death is identified. Despite this, fetal growth restriction remains the most frequent primary cause of death within the study population, and re-classified deaths are principally attributed to placental insufficiency (the principal cause of fetal growth restriction).
